# Genetic Risk Factors for Nontuberculous Mycobacterial Pulmonary Disease (Systematic Review)

**DOI:** 10.17691/stm2024.16.5.07

**Published:** 2024-10-30

**Authors:** P.S. Sviridov, M.M. Litvinova, M.A. Karnaushkina, N.N. Makaryants, M.V. Gorbunova

**Affiliations:** Laboratory Geneticist, Researcher; Research Centre for Medical Genetics, 1 Moskvorechye St., Moscow, 115522, Russia; Research Assistant; The Patrice Lumumba Peoples’ Friendship University of Russia, 6 Miklukho-Maklaya St., Moscow, 117198, Russia; MD, PhD, Associate Professor, Deputy Head of the Department for Research Work of the Department of Medical Genetics; I.M. Sechenov First Moscow State Medical University (Sechenov University), 8/2 Trubetskaya St., Moscow, 119991, Russia; Senior Researcher; The Patrice Lumumba Peoples’ Friendship University of Russia, 6 Miklukho-Maklaya St., Moscow, 117198, Russia; MD, DSc, Professor, Department of Internal Medicine with the Course of Cardiology and Functional Diagnostics named after Academician VS. Moiseyev; The Patrice Lumumba Peoples’ Friendship University of Russia, 6 Miklukho-Maklaya St., Moscow, 117198, Russia; MD, DSc, Leading Researcher; Head of the Department of Differential Diagnosis of Tuberculosis and Exstracorporeal Methods of Treatment; Central Scientific Research Institute of Tuberculosis, 2 Yauzskaya Alleya, Moscow, 107564, Russia; Leading Researcher; The Patrice Lumumba Peoples’ Friendship University of Russia, 6 Miklukho-Maklaya St., Moscow, 117198, Russia; MD, DSc, Associate Professor, Department of Phthisiatry and Pulmonology, Educational Research Institute for Clinical Medicine named after N.A. Semashko; Russian University of Medicine, 3 Rakhmanovsky Pereulok, Moscow, 127994, Russia

**Keywords:** nontuberculous mycobacterial pulmonary disease, NTM-PD, genetic markers, polymorphism, immunity, risk factors

## Abstract

This paper is a systematic review of the published data describing genetic risk factors for pulmonary diseases caused by nontuberculous mycobacteria (nontuberculous mycobacterial pulmonary disease — NTM-PD).

**The aim of the study** is to compile a specific list of genetic markers associated with the risk of developing NTM-PD.

This literature review was prepared according to PRISMA criteria and was registered in the International Prospective Register of Systematic Reviews (PROSPERO) (registration number CRD42019128569).

In the process of work, a great number of articles from PubMed, Google Scholar, and ScienceDirect databases have been studied. Using careful analysis and selection procedures, a list of 14 genetic variants associated with an increased risk of developing NTM-PD was generated. *SLC11A1, NLRP3, TLR2, CFTR, IFNGR1, PDCD1* genes have been found to refer to these variants as well as variants in the intergenic regions affecting expression of *STK17A, IFNL3, TNF, IL10* genes. The products of these genes take different roles in regulating the response to various pathogenic factors, and some of them are poorly understood. For a more precise and detailed explanation of the influence of these genetic variants, further studies in patient groups of different populations with the evaluation of different combinations of variants and intergenic interaction are required.

## Introduction

The genus *Mycobacterium* is a large heterogeneous group of microorganisms including saprophytic and opportunistic species, some of which can cause such diseases as tuberculosis, leprosy, and mycobacterioses [1, 2]. The ubiquitous spread of infections associated with nontuberculous mycobacteria (NTM) (Table 1) [3–8] has provoked increased interest of the medical community in the diseases caused by these pathogens [1, 9, 10].

**T a b l e 1:** Prevalence of infections caused by nontuberculous mycobacteria in some countries

Country (years)	Incidence (per 100,000 population)	References
Great Britain, 1995–2006	0.9–2.9	Moore et al. [3]
Canada, 1998–2010	4.5–9.08	Brode et al. [4]
Taiwan, 2000–2008	2.65–10.17	Lai et al. [5]
South Korea, 2003–2016	1.2–33.3	Parket al. [6]
USA, 2008–2013	9.2–15.2	Donohue and Wymer [7]
Russia, 2016	1.5–4.3	Beloborodova et al. [8]

The growing number of identified NTM-associated infections may be connected with both the improvements in diagnostic methods and with the increased awareness of these mycobacteria among medical specialists [3, 5]. However, diseases referred to NTM infections including the more common form, nontuberculous mycobacterial pulmonary disease (NTM-PD), are not in the list for mandatory registration [9]. This makes it difficult to accurately calculate the actual incidence rate.

Immunocompromised individuals suffering from HIV infections; patients receiving immunosuppressive therapy after organ transplantation; patients with chronic pulmonary diseases such as cystic fibrosis, chronic obstructive pulmonary disease, bronchiectasis, and others, are at higher risk of NTM-PD development. Elderly people are also susceptible to a high risk of NTM-PD development [11, 12].

Therefore, identifying genetically determined factors that predispose individuals to NTM-PD may aid in developing personalized approaches to prevent complications in at-risk individuals.

**The aim of the systematic review** is to compile a list of genetic markers causing an increased risk of developing nontuberculous mycobacterial pulmonary disease based on the analysis of the literature sources and genome databases.

## Materials and Methods

***The inclusion and exclusion criteria*** are presented in Table 2. Case-control studies and genome-wide association studies (GWAS) were selected for analysis. The examined groups consisted of individuals over 18 years, excluding patients with HIV, cancer, or those with systemic diseases receiving immunosuppressive therapy. The described genetic variant was considered protective if the odds ratio (OR) was less than 1 (OR<1). In the case of OR=1, the correlation was not found. At OR>1 the association between the genetic variant and increased risk of NTM-PD development was established. The association was considered statistically significant only at p≤0.05. Therefore, we excluded literature sources where no association was detected or where the OR was ≤1.

**T a b l e 2:** Criteria of inclusion and exclusion of the literature sources

Inclusion criteria	Exclusion criteria
Cohort	Persons under 18 years
GWAS	Oncological patients
Persons over 18 years	HIV infected
Odds ratio >1	Persons with systemic diseases receiving immunosuppressive therapy
Statistical significance (p≤0.05)	Odds ratio ≤1
	Insufficient statistical significance (p>0.05)

### The literature search strategy

For our work, we used three global databases: PubMed, Google Scholar, and ScienceDirect. Searching was not limited by the date of publication.

The search query in PubMed included phrases “nontuberculous mycobacterial lung disease” or “nontuberculous mycobacterial pulmonary disease” and also the words “gene”, “polymorphism”, and their derivatives. The output information of the pre-filtered search results should not contain the word “children” and its derivatives. In compliance with the above conditions, the query for the search algorithm looked as follows:

(((“nontuberculous”[All Fields] AND (“mycobacterial” [All Fields] OR “mycobacterials”[All Fields]) AND (“lung diseases”[MeSH Terms] OR (“lung”[All Fields] AND “diseases”[All Fields]) OR “lung diseases”[All Fields] OR (“lung”[All Fields] AND “disease”[All Fields]) OR “lung disease”[All Fields])) OR (“nontuberculous”[All Fields] AND (“mycobacterial”[All Fields] OR “mycobacterials”[All Fields]) AND (“lung diseases”[MeSH Terms] OR (“lung”[All Fields] AND “diseases”[All Fields]) OR “lung diseases”[All Fields] OR (“pulmonary”[All Fields] AND “disease”[All Fields]) OR “pulmonary disease”[All Fields]))) AND (“genes”[MeSH Terms] OR “genes”[All Fields] OR “gene”[All Fields]) AND (“polymorphic”[All Fields] OR “polymorphics”[All Fields] OR “polymorphism s”[All Fields] OR “polymorphism, genetic”[MeSH Terms] OR (“polymorphism”[All Fields] AND “genetic”[All Fields]) OR “genetic polymorphism”[All Fields] OR “polymorphism”[All Fields] OR “polymorphisms”[All Fields])) NOT (“child”[MeSH Terms] OR “child”[All Fields] OR “children”[All Fields] OR “child s”[All Fields] OR “children s”[All Fields] OR “childrens”[All Fields] OR “childs”[All Fields]).

As a result of our search, 28 publications were selected. Due to the limited number of sources, additional searches were conducted in the Google Scholar and ScienceDirect databases. After inputting the phrase “genetic factors of nontuberculous mycobacterial lung disease” as a query in Google Scholar, 25,700 records were obtained. In the ScienceDirect search field, we entered “nontuberculous mycobacterial pulmonary disease genetics” and obtained 348 records. The papers were then screened by title and abstract to exclude articles not suitable for the topic of our work. Studies on mycobacterial cultures, clinical observations, and duplicate entries were excluded from the article selection. Eventually, the list of literature sources contained 42 articles.

At the next stage, the full texts of the selected articles and their accompanying materials (if any) were thoroughly analyzed according to the criteria of inclusion and exclusion. Articles, in which the tested group consisted of individuals under 18 years, patients with oncological diseases or individuals with systemic diseases receiving immunosuppressive therapy, were excluded. Papers, in which the association between the genetic variants and the risk of NTM-PD development was statistically insignificant (p>0.05), were also excluded.

**Figure 1.**
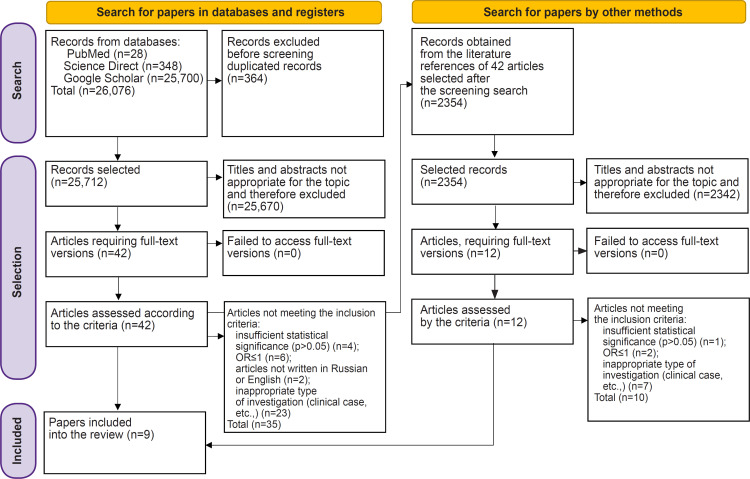
The scheme of data search using the PRISMA platform

Additionally we reviewed the reference lists in the selected articles and analyzed 12 additional publications. After excluding articles that did not meet our criteria, 9 papers remained. The scheme of source selection is presented in Figure 1.

This literature review was prepared according to PRISMA criteria and was registered in the International Prospective Register of Systematic Reviews (PROSPERO) (registration number CRD42019128569).

## Results

As a result of careful selection and systematic analysis of numerous publications, a list of genetic markers, which may be indicative of a high risk for NTM-PD development was compiled. This list includes 14 genetic variants in 10 genes (Table 3) [13–20]. These genetic variants are associated with various immunological processes including the response to mycobacteria. Information on the role of each gene and its product is presented below.

**T a b l e 3:** Genetic variants associated with a high risk for the development of nontuberculous mycobacterial pulmonary disease

No	Gene	Genetic variant (GRCh38)	Number in dbSNP database	Tested allele or genotype, OR of the risk variant, CI, and p-value	Type of investigation and samples	NTM-PD caused by mycobacteria	References
1	*SLC11A1* (also known as *NRAMP1*, *NRAMP*)	c.*56_*59del	rs17235416	Genotype TGTG+/del: OR=9.54, 95% CI: 2.49–36.53, p<0.001	Cohort study Koreans, patients — 41, healthy — 50	*M. avium-intracellulare* complex (n=18), *M. abscessus* (n=23)	Koh et al. [13]
2		c.1627G>A, p.(Asp543Asn)	rs17235409	Genotype G/A: OR=5.74, 95% CI: 1.48–22.30, p=0.006			
3		c.393+14G>C	rs3731865	Genotype G/C: OR=2.78, 95% CI: 1.12–6.89, p=0.026			
4	*NLRP*3	c.1302C>T, p.(Ser434=)	rs34298354	Women with C/T genotype: adjOR=8.517, 95% CI: 1.010– 71.838, p=0.049	Cohort study Taiwanese, patients — 106, healthy — 119	*M. avium-intracellulare* complex	Wu et al. [14]
5	*TLR2*	c.1350T>C, p.(Ser450=)	rs3804100	Men with T/C genotype: adjOR=3.050, 95% CI: 1.218– 7.638, p=0.017			
6		≤(GT)16 (S-allele)	No data	All patients with NTM-PD: OR=1.70, 95% CI: 1.11–2.61, p=0.01 *M. avium-intracellulare* complex: OR=1.91, 95% CI: 1.16–3.16, p=0.01	Cohort study Koreans, patients — 193, healthy — 191	*M. avium-intracellulare* complex (n=110), *M. abscessus* (n=82), *M. intracellulare* and *M. abscessus* (n=1)	Yim et al. [15]
7	*CFTR*	c.4056G>C, p.(Gln1352His)	rs113857788	Genotype G/C: OR=4.27, 95% CI: 1.43–12.78, p<0.001 Allele C: OR=4.10, 95% CI: 1.39–12.13, p<0.001	Cohort study Koreans, patients — 360, healthy — 446	*M. avium-intracellulare* complex (n=249), *M. abscessus* complex (*M. abscessus*, *M. massiliense*) (n=111)	Jang et al. [16]
8	*IFNGR1*	c.-56T>C	rs2234711	C allele (was in 70% of persons with NTM-PD): OR=0.6, 95% CI: 0.4-0.9, p<0.05 Genotype T/C: OR=1.3, 95% CI: 0.5-1.8, p<0.05 Genotype C/C: OR=1.5, 95% CI: 1.4-1.7, p<0.05	Cohort study Iranians, patients — 80, healthy — 80	*M. simiae* (n=62), *M. kansassii* (n=4), *M. avium-intracellulare complex* (n=3), *M. chelonae* (n=6), *M. fortuitum* (n=5)	Farnia et al. [17]
9	Intergenic variant regulating expression of *STK17A* (also known as *DRAK1*)	g.43744189T>C	rs849177	All patients with NTM-PD: OR=2.34, 95% CI: 1.71-3.21, p=1.36·10-^7^ *M. avium-intracellulare* complex: OR=2.40, 95% CI: 1.71-3.37, p=4.30·10-^7^ *M. abscessus* complex: OR=2.14, 95% CI: 1.27-3.61, p=4.21·10-^3^	GWAS Koreans, patients — 412, healthy — 1056 (from 384 families)	*M. avium-intracellulare* complex, *M. abscessus* complex	Cho et al. [18]
10	*PDCD1*	c.644C>T, p.(Ala215Val)	rs2227982	High risk for men — genotype G/G vs A/G vs A/A: OR=2.205, 95% CI: 1.108-4.389, p=0.02	Cohort study Taiwanese, patients — 152 (97 women), healthy — 1056 (85 women)	*M. avium-intracellulare* complex	Pan et al. [19]
11		c.*889G>A	rs10204525	Lower risk for women — genotype T/T vs T/C vs C/C: OR=0.396, 95% CI: 0.176-0.890, p=0.02			
12	Intergenic variant regulating expression of *IFNL3* (also known as *IL28B*)	g.39252525T>G	rs8099917	Genotype T/G: OR=2.2, 95% CI: 1.17–4.20, p=0.01 (multivariate analysis)	Cohort study Australians, patients — 79, healthy — 188	*M. intracellulare*, *M. avium*	Affandi et al. [20]
13	Intergenic variant regulating expression of *TNF*	g.31574531T>C	rs1799964	Carriage of C/T genotype in comparison with C/C genotype reduced the probability of NTM-PD development: OR=0.50, 95% CI: 0.3–0.9, p=0.03 — univariate analysis; OR=0.48, 95% CI: 0.25–0.93, p=0.02 — multivariate analysis			
14	Intergenic variant regulating expression of *IL10*	g.206773552T>C	rs1800896	Carriage of A/G genotype in comparison with A/A genotype reduced the probability of NTM-PD development: OR=0.40, 95% CI: 0.2–0.7, p=0.004 — univariate analysis; OR=0.33, 95% CI: 0.17–0.65, p=0.001 multivariate analysis			

N o t e: NTM-PD — nontuberculous mycobacterial pulmonary disease, OR — odds ratio, CI — confidence interval.

The *SLC11A1* gene (also *NRAMP*, *NRAMP1*), encoding the similarly named protein, is located on the long arm of chromosome 2 (2q35). SLC11A1 protein is an analog of the mouse Slc11a1 protein, which is involved in the protection against microorganisms including *Mycobacterium tuberculosis*, leishmania, and salmonella [21–23]. The primary function of this protein is to transport metal ions in any direction against the proton gradient. SLC11A1 is located on lysosomal membranes and transports divalent cations from the cytosol into lysosomes for their direct participation in antimicrobial activity. Separation of metal ions, Fe^2+^ and Mn^2+^ in particular, being cofactors in prokaryotes and eukaryotes, promotes protection of macrophages against reactive oxygen species and simultaneously does not allow pathogenic microorganisms to use iron and magnesium for synthesis of protective enzymes [24, 25].

The *NLRP3* gene, which encodes the NLRP3 protein, is located on the long arm of chromosome 1 (1q44). This protein, also known as cryopirin, expresses mainly in chondrocytes and white blood cells, in macrophages in particular. Cryopirin is part of the NLRP3 inflammasome and is responsible for its activation in response to cell membrane damage and to the presence of foreign bodies [26]. Activation of NLRP3 is stimulated by extracellular adenosine triphosphate, nigericin, reactive oxygen species, crystals of urates or cholesterol, beta-amyloid fibers, as well as particles and nanoparticles such as asbestos, silicon oxide, coal dust, and others [27–29].

It should be noted that Wu et al. [14] have found an association of rs34298354 variant with the development of NTM-PD caused by the *Mycobacterium avium* complex in women with C/T genotype (adjOR=8.517; p=0.049). The authors also found a possible association of the rs3806268 variant with the development of NTM-PD. Thus, OR for G/A genotype of polymorphic locus rs3806268 was 1.9 (adjOR=1.945; p=0.085). However, the rs3806268 variant was not included in our list because the value of p was higher than 0.05.

The *TLR2* gene encodes a protein of the same name, which is a member of the toll-like receptor (TLR) family and plays a fundamental role in recognizing pathogens and activating innate immunity. TLR2 is located on the cell surface and may form heterodimers with other proteins of the TLR family to recognize pathogen-associated molecular patterns (PAMPs). Activation by PAMPs triggers signaling pathways and subsequent inflammatory response. This protein is also considered to promote apoptosis in response to bacterial lipoproteins found in *M. tuberculosis* [30–32]. TLR2 is associated with the pathogenesis of several autoimmune diseases.

The *CFTR* gene, encoding the CFTR protein, is located on the long arm of chromosome 7 (7q31.2). CFTR is a transmembrane regulator of cystic fibrosis and is expressed in various organs, including the pancreas, lungs, ducts of the perspiratory glands, and other tissues [33–35]. These proteins belong to the ABC transporter family and consist of two transmembrane domains connected by cytoplasmic domains. Incorporating into the apical membrane of the epithelial cells, CFTR regulates transport of chloride ions [36]. Damage to the chloride channel results in changes in the cell electrolyte composition and, as a consequence, in the impairment of the secreted product [37].

The *IFNGR1* gene is located on the long arm of chromosome 6 (6q23.3) and encodes the IFNGR1 protein. This protein, along with IFNGR2, is part of the interferon-gamma receptor, which plays an important role in antimicrobial and antitumor responses activating immune effector cells and enhancing antigen presentation [38, 39].

The *STK17A* gene (also known as *DRAK1*) is located on the short arm of chromosome 7 (7p13) and encodes the STK17A protein, which belongs to serine/ threonine kinases. This protein is involved in regulation of apoptosis and reactive oxygen species metabolism [40, 41].

The *PDCD1* gene is located on the long arm of chromosome 2 (2q37.3) and encodes the PD1 membrane protein, which belongs to the immunoglobulin superfamily. PD1 binds two ligands, PD-L1 and PD-L2, and participates in the negative regulation of the immune system preventing activation of T lymphocytes, thereby reducing autoimmunity and increasing concurrently autotolerance [42]. The *PDCD1*-mediated pathway may be used by cancer cells to change the antitumor response and avoid the destruction by the immune system [43].

The *IFNL3* (also known as *IL28B*) is found on the long arm of chromosome 19 (19q13.2) and encodes interferon lambda 3 protein, a cytokine with antiviral, antitumor, and immunomodulatory activities. This protein plays an important role in the antiviral response (mainly in the epithelial tissues), being a ligand for a heterodimeric receptor consisting of IL10RB and IFNLR1. The interaction with the receptor results in activation of the JAK/STAT pathway and expression of the interferon-stimulated genes [44, 45].

The *TNF* gene, encoding tumor necrosis factor alpha (TNF-α), is on the short arm of chromosome 6 (6p21.33). TNF-α is a cytokine, secreted by macrophages, which facilitates cell death of certain tumor cell lines. This cytokine is also an important pyrogene, inducing fever, acting directly or stimulating secretion of interleukin-1. Under some conditions, TNF-α may facilitate cell proliferation and induce cell differentiation [46].

The *IL10* gene is located on the long arm of chromosome 1 (1q32.1) and encodes interleukin-10, a cytokine, which is involved in limiting immune response and infammation, helping reduce injury to the cells in the body. Mutations in this gene are associated with susceptibility to HIV infection and rheumatoid arthritis [47–49].

## Discussion

According to data from the analyzed articles, a high risk for NTM-PD development is associated with female gender, chronic pathology of respiratory organs, and decreased immunological reactivity [50]. A genetic factor is a nuance, which, if combined even with one of the above factors, may play a decisive role in the development of an active pathological process and determine its course. Given the connection of NTM-PD with the conditions that reduce the body’s defenses against foreign antigens, people with the mentioned risk factors may be assumed to have genetically determined features of innate immunity, just as it has been demonstrated in our review.

**Figure 2.**
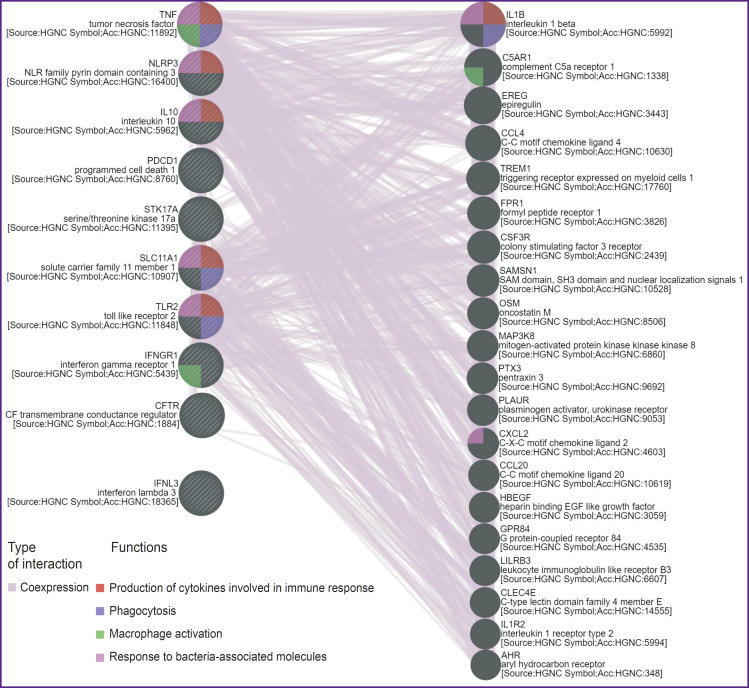
The scheme of coexpression of genes associated with a high risk for developing nontuberculous mycobacterial pulmonary disease (image was generated using GeneMANIA server [51])

Based on the presented information, it may be suggested that at least 10 genes and their polymorphisms are associated with the development of NTM-PD. The list of genes presented in Table 3 may be divided according to the functions of proteins encoded by them. The products of *IFNL3*, *TLR2*, *IFNGR1*, *PDCD1*, *IL10* genes are regulatory proteins participating in enhancement or inhibition of some immune reactions; proteins encoded by *SLC11A1* and *CFTR* genes may be referred to transport genes providing movement of certain molecules depending on the cell needs; *NLRP3* gene encodes the inflammatory response-related protein, which is part of the inflammasome complex; products of *STK17A*, *TNF* genes are proteins engaged in triggering controlled cell death. Among the genes indicated in this article, those involved in forming the response to bacterial molecules prevail in the variants associated with a high NTM-PD risk (Figure 2; image was generated using GeneMANIA server [51]).

Despite careful selection and analysis of the literature, the list includes genes responsible for synthesizing multifunctional, often non-specific factors, such as cytokines. This reduces the reliability of interpreting their role as predictors of NTM-PD development and requires further, more in-depth exploration.

Additionally, each of the presented polymorphisms does not act in isolation but contributes to the risk of disease development in the context of the patient’s overall genetic constitution. In this context, special attention should be paid to studying the interactions between genetic factors and investigating the role of combinations of receptor gene polymorphisms and opsonizing proteins involved in innate immune responses. Additionally, analyzing the interconnections of gene variants coexpressed with those associated with NTM-PD development in our study is of great interest (see Figure 2).

The association of genetic variants with NTM-PD has been demonstrated in different populations. Therefore, the frequency and contribution of each of the mentioned genetic marker may differ among the representatives of these populations. This emphasizes the importance of studying the relationship between these polymorphisms and the risk of NTM-PD development in the Russian population.

Analyzing the literature sources, we did not find data on genetic features promoting preferential development of the infectious process caused by a specific bacterial species. In the published investigations, the most commonly diagnosed mycobacterial complexes are described (primarily *M. avium-intracellulare* complex) — about 50% of all NTM-PD [50, 52]. It is likely to be connected with the fact that diseases caused by separate strains of NTM-PD are encountered in a small percentage of cases, which makes it difficult to obtain statistically significant results [53].

Thus, the effect of genetic factors on NTM-PD morbidity and its course, depending on the causative agent, requires further exploration.

## Conclusion

Through our analysis of the literature, we identified 14 genetic variants in 10 human genes that are most likely associated with an increased risk of NTM-PD development. Further studies are needed to examine the effect of these variants on NTM-PD morbidity in various populations and to investigate potential interactions between the genes. This will require the development of molecular and genetic systems to detect specific genetic variants. Additionally, determining the frequency of these risk alleles in the Russian population is the next important step. The molecular effects of these variants should also be studied in a large sample of patients with pulmonary diseases caused by nontuberculous mycobacteria to determine the true impact of genetic variants on the risk of NTM-PD morbidity.
